# Evaluation of the Immune Response Induced by CoronaVac 28-Day Schedule Vaccination in a Healthy Population Group

**DOI:** 10.3389/fimmu.2021.766278

**Published:** 2022-01-31

**Authors:** Alejandro Escobar, Felipe E. Reyes-López, Mónica L. Acevedo, Luis Alonso-Palomares, Fernando Valiente-Echeverría, Ricardo Soto-Rifo, Hugo Portillo, Jimena Gatica, Ivan Flores, Estefanía Nova-Lamperti, Carlos Barrera-Avalos, María Rosa Bono, Leonardo Vargas, Valeska Simon, Elias Leiva-Salcedo, Cecilia Vial, Juan Hormazabal, Lina Jimena Cortes, Daniel Valdés, Ana M. Sandino, Mónica Imarai, Claudio Acuña-Castillo

**Affiliations:** ^1^ Laboratorio Biología Celular y Molecular, Instituto de Investigación en Ciencias Odontológicas, Facultad de Odontología, Universidad de Chile, Santiago, Chile; ^2^ Centro de Biotecnología Acuícola, Facultad de Química y Biología, Universidad de Santiago de Chile, Santiago, Chile; ^3^ Department of Cell Biology, Physiology and Immunology, Universitat Autònoma de Barcelona, Bellaterra, Spain; ^4^ Facultad de Medicina Veterinaria y Agronomía, Universidad de Las Américas, Providencia, Chile; ^5^ Laboratory of Molecular and Cellular Virology, Virology Program, Institute of Biomedical Sciences, Faculty of Medicine, Universidad de Chile, Santiago, Chile; ^6^ Molecular and Translational Immunology Laboratory, Department of Clinical Biochemistry and Immunology, Faculty of Pharmacy, University of Concepcion, Concepcion, Chile; ^7^ Departamento de Biología, Facultad de Ciencias, Universidad de Chile, Santiago, Chile; ^8^ Programa Hantavirus y Zoonosis, Instituto de Ciencias e Innovación en Medicina, Facultad de Medicina, Clínica Alemana Universidad del Desarrollo, Santiago, Chile

**Keywords:** CoronaVac, SARS-CoV-2, herd immunity, neutralizing antibodies, COVID-19, vaccine, immunological memory

## Abstract

CoronaVac vaccine from Sinovac Life Science is currently being used in several countries. In Chile, the effectiveness of preventing hospitalization is higher than 80% with a vaccination schedule. However, to date, there are no data about immune response induction or specific memory. For this reason, we recruited 15 volunteers without previous suspected/diagnosed COVID-19 and with negative PCR over time to evaluate the immune response to CoronaVac 28 and 90 days after the second immunization (dpi). The CoronaVac administration induces total and neutralizing anti-spike antibodies in all vaccinated volunteers at 28 and 90 dpi. Furthermore, using ELISpot analysis to assay cellular immune responses against SARS-CoV-2 spike protein, we found an increase in IFN-gamma- and Granzyme B-producing cells in vaccinated volunteers at 28 and 90 dpi. Together, our results indicate that CoronaVac induces a robust humoral immune response and cellular immune memory of at least 90 dpi.

## Introduction

The pandemic of coronavirus disease 2019 (COVID-19) has led to an unprecedent rapid vaccine development and production aimed to prevent the spread of the SARS-CoV-2 virus. The clinical severity of the disease has led to the urgent application and approval of vaccines with phase 3 studies under development, with proven safety results, but with little data regarding immune response, efficacy, and mechanism of action to prevent the disease. In this scenario, the immune mechanisms triggered by SARS-CoV-2 vaccines in healthy populations are not well-known. Studies are limited, especially from laboratories not directly related to the manufacture of vaccines or research teams leading the evaluations of phase 2 or 3 clinical trials (1).

Currently, in Chile, two types of vaccines have been massively applied, the BNT162b2 vaccine by Pfizer-BioNTech vaccine generated from engineered messenger RNA (mRNA) encoding the spike protein (S) of SARS-CoV-2 (2), and the CoronaVac vaccine from Sinovac Life Sciences that contains the inactivated SARS-CoV-2 virus (3). A recent report in the Chilean population vaccinated with two doses of CoronaVac separated by 14 days showed a seroconversion rate for anti-S1-RBD (receptor binding domain) IgG of 18.1%, 100%, and 87.5% at 14, 28, and 42 days post-immunization (dpi), respectively. Moreover, a 95.7% seroconversion rate was reported for neutralizing antibodies in the 18- to 59-year-old group after 28 and 42 dpi, and the induction of a T-cell response characterized by the secretion of interferon (IFN)-gamma (IFN-γ) upon stimulation with SARS-CoV-2 Mega Pools of peptides was also observed (4).

The current COVID-19 mass vaccination schedule consists of two doses of the vaccines separated by 4 weeks (28 days apart schedule vaccination) (5). Recently, a prospective national cohort study reported that using this vaccination schedule, CoronaVac is effective for preventing hospitalization (87.5%), Intensive Care Unit (ICU) admission (90.3%), and Covid-19–related death (86.3%) (6). Many questions have arisen about the need of a third booster dose, especially given the absence of detailed and conclusive evidence of the immune responses induced by CoronaVac using the 28-day-apart vaccination. Therefore, in this study, we evaluated the production of neutralizing antibodies, the activation of the cellular response, and the generation of cellular memory induced after CoronaVac 28-day schedule vaccination in a healthy population group independently of the production laboratory. Together, our results indicate that CoronaVac induces a robust humoral immune response and cellular immunity of at least 90 dpi, which can explain prevention of COVID19 with severe symptoms (6, 7).

## Materials and Methods

### Study Population, Study Design, and Statistical Analysis

Twenty-one adult volunteers (mean age, 36 years old [range 27 to 61]) who were scheduled to receive the CoronaVac vaccine were recruited from professionals from the area of clinical laboratories for COVID detection at the Universidad de Santiago de Chile during March 2021. The sample size was calculated according to the associated hypothesis using the G test. Since a 60% protection has been described for this vaccine, a 60% induction of memory at 90 days post-vaccination was expected. This analysis showed that the minimum sample size was 15 volunteers. Because usually, volunteers declined their participation without completing the studies, about 30 volunteers were recruited. Inclusion criteria included the absence of suspected/diagnosed case of COVID-19 and testing negative for COVID-19 for at least six previous PCR analyses in monthly screening tests. In addition, immunological diseases or any associated chronic disease with no treatment were considered exclusion criteria. Blood samples were obtained before the vaccination process. The Scientific Ethics Committee approved the study and informed consent, Universidad de Santiago de Chile (172/2021). In addition, written informed consent was obtained from each enrolled participant. Paired samples were analyzed using Friedman and Quade test with multiple comparison post-test. Unpaired data analysis was performed using Mann–Whitney test. All statistical analyses were accomplished with GraphPad Prism version 8.01 (GraphPad Software Inc, La Jolla, CA). The results were presented as median. *p* < 0.05 was considered statistically significant.

### Sample Processing

Blood was collected in BD Vacutainer tubes (REF367281, Becton, Dickinson and Company, USA) by health-trained personnel. The samples obtained (15 ml at each time point) were coded, kept cold, and processed to obtain plasma and peripheral blood mononuclear cells (PBMCs). One milliliter of blood was aliquoted to obtain blood plasma by centrifugation. Then, 300 µl was transferred to cryopreservation tubes and frozen at −80°C until use. White cells were isolated by Ficoll-Hypaque gradient centrifugation (Gibco BRL lymphoprep).

### Production and Purification Spike Protein from SARS-CoV-2

The sequence coding for the spike protein modified by Amanat et al. (GenBank: MN908947.3) was commercially synthesized and cloned into the pcDNA3.1 plasmid (GenScript, Piscataway, NJ, USA). The transfection was performed using the ExpiCHO Expression System. Briefly, the ExpiCHO-S cells were cultured in a vented flask with ExpiCHO Expression medium (Thermo Fisher, Cat. A2910001) in a humidified 8% CO_2_ incubator at 37°C at 400*g* and passaged every 3–4 days at 200,000 cells/ml, until transfection day. The day before transfection, the cells were split at 3 × 10^6^ cells/ml. After 24 h, the cells were diluted at 6 × 10^6^ cells/ml with a fresh medium. The ExpiCHO transfections were performed using the ExpiCHO Expression System Kit (Thermo Fisher, Cat. A29133), according to the manufacturer’s protocol, and cultures were then temperature shifted to 32°C and cultured for 5 to 7 days. Then, cell suspension was centrifuged at 3,220*g* for 20 min at 4°C to pellet cells. The supernatant was collected and centrifuged at 5,000*g* for 30 min, the pH was adjusted to 8.0, and inhibitor proteases were added. The supernatant was dialyzed in native dialysis buffer (50 mM Tris–HCl, pH 8.0, 250 mM NaCl) overnight at 4°C. The purification was performed by exclusion chromatography in Ni-NTA (Gold Bio, Cat H-350-100). Briefly, columns were equilibrated using native binding buffer (50 mM Tris–HCl, pH 8.0, 250 mM NaCl, 10 mM Imidazole). The supernatant was loaded onto the equilibrated columns and incubated for 2 h at 4°C in an orbital shaker. Following incubation, the supernatant was collected, and the columns were washed twice with native wash buffer (50 mM Tris–HCl, pH 8.0, 250 mM NaCl, 20 mM Imidazole). Next, the protein was eluted in the elution buffer (50 mM Tris–HCl, pH 8.0, 250 mM NaCl, 250 mM Imidazole) and concentrated in an Amicon Ultra-15 centrifugal filter unit (50,000 NMVL) at 3,220*g* at 4°C for 15 min. The buffer was discarded, and the protein was washed twice using storage buffer (20 mM Tris–HCl, pH 8.0, 150 mM NaCl). Proteins were analyzed by reducing SDS-PAGE, dyeing with Sypro Ruby (Thermo Fisher, Cat. S12001), and quantified using ImageJ software. Finally, the Spike protein was aliquoted and stored at −80°C, until use.

### Cytokines

Analyses were performed in plasma samples obtained from the volunteers, before vaccination and 28 days after the second immunization. The cytokines TNF-α, IL-12, IL-6, IL-10, IL-1β, and IL-8 were quantified using the Cytometric Bead Array (CBA) System (Becton Dickinson, BD-USA). Data acquisition was achieved by flow cytometry using a FacsCanto II flow cytometer (BD-USA) and analyzed in the BD FCAP Array software, where values were expressed in pg/ml for each cytokine.

### Total Anti-Spike Antibodies

Total antibody levels were determined using a previously established protocol (https://doi.org/10.1038/s41591-020-0913-5). Briefly, ninety-six-well plates were coated at 4°C with 50 ng/well of the recombinant SARS-COV-2 S protein S1 (Biolegend 792906) in PBS. After overnight incubation, the coating solution was removed and 100 μl/well of PBS, containing 0.1% Tween-20 (PBST) and 3% non-fat milk, were added to the plates. Plates were incubated for 1 h at room temperature. The serum samples were previously heated at 56°C for 1 h, and serial dilutions of serum were prepared in 1% non-fat milk prepared in PBST. One hundred microliters of each serial dilution was added, and the plates were incubated for 2 h at room temperature. The plates were washed three times using 0.1% PBST and then 100 μl of a 1:10,000 dilution of donkey anti-human IgG–horseradish peroxidase (HRP) conjugated antibody (Clone Poly24109, Biolegend 410902) was added to each well for 1 h. Plates were again washed with 0.1% PBST and then dried. Next, 100 μl of TMB (3,3′,5,5′-Tetramethylbenzidine BD OptEIA) solution was added to each well. This substrate was left on the plates for 10 min and then the reaction was stopped by the addition of 50 μl per well of 1 M Phosphoric acid. The optical density at 490 nm was measured using an Emax (Molecular Devices, USA) plate reader. Results are calculated as the area under the curve of four-serial dilutions of patient plasma obtained by the ELISA technique.

### IgM and IgG

Rapid tests (OnSite Covid-19 IgG/IgM Rapid Test-CTK Biotech, Inc, USA) were used according to manufacturer’s instructions to detect antibodies before vaccination, and at 28, 48, 78, 108, and 138 dpi.

### Neutralizing Antibodies

Pseudotyped viral particles were produced by transient transfection of HEK293T cells using polyethylenimine (PEI) and plasmids pNL4.3-ΔEnv-Firefly and pCMV14–3X-Flag–SARS-CoV-2 SΔ19C in a 1:1 ratio as we recently described (8). Cell culture media was replaced and collected at 16 h and 48 h post-transfection, respectively, and centrifugated to 850*g* for 5 min at room temperature. Viral particles were diluted with 50% in fetal bovine serum (Sigma-Aldrich) and stored at −80°C. Viral stock was quantified with the HIV-1 Gag p24 Quantikine ELISA kit (R&D Systems) and titrated by serial dilutions. Neutralization assays were performed as we previously reported (8). Briefly, inactivated serum samples were diluted in DMEM with 10% fetal bovine serum and incubated with 3 to 5 ng of p24 of HIV-1-based SARS-CoV-2 pseudotyped particles. After 1 h incubation at 37°C, 1 × 10^4^ HEK-ACE2 cells were added to each well. HEK293T cells incubated with the pseudotyped virus were used as a negative control. Cells were lysed 48 h later, and firefly luciferase activity was measured using the Luciferase Assay Reagent (Promega) in a Glomax 96 Microplate luminometer (Promega). The percentage of neutralization for each dilution was calculated, and neutralization titers, defined as 50% inhibitory dose (ID50), were estimated as previously described (8). Briefly, ID50 for each sample was obtained using a four-parameter non-linear regression. Pass/fail quality control criteria for the assay were as follows (1): the average RLUs of pseudotype control wells are ≥10 times the average RLUs of negative control (2); the % coefficient of variation (CV) of RLUs for triplicate wells control is ≤30% (3); the % CV of duplicate is ≤30% for sample dilutions that yield at least 40% neutralization; and (4) positive control neutralization curve crosses the 50% neutralization cutoff 0 to 1 times. All statistical analyses were performed using GraphPad Prism version 8.0.1 software.

### Enzyme-Linked Immunospot

The specific activation of T lymphocytes was evaluated by IFN-γ and Granzyme B kit (R&D system). PBMCs were isolated by using Ficoll-Hypaque gradient centrifugation (Lymphoprep Gibco BRL) and leucocyte cell numbers were quantified using Trypan Blue. Anti–IFN-γ and Anti-Human Granzyme B antibody-coated wells (R&D Systems) were seeded with 300,000 leucocyte cells per well. Cells were stimulated in duplicate with 10 µM SARS-CoV-2 spike structural protein (1.3 μg/ml). OKT3 and CD28 were used as positive control of stimulation (3 and 5 μg/ml, respectively). Culture medium was the negative control. PBMCs were cultured for 48 h before enzymatic revelation of IFN-γ and Human Granzyme B (R&D Systems). Spots were counted by using an ELISPOT reader (A.EL.VIS ELISPOT Scanner) and results are expressed as the mean number of spot-forming units/3 × 10^6^ peripheral blood mononuclear cells after subtraction of the background value.

## Results

### Characteristics of the Study Population

We evaluated the immune response to the CoronaVac vaccine applied in the Chilean population following an interval of 28 days between the first and second dose schedule. We measured the capability of the vaccine to activate the humoral and cellular responses in volunteers without suspected/diagnosed COVID-19 and with negative PCR over time throughout the whole study. **Table 1** shows the demographic and baseline characteristics of the enrolled volunteers. We assessed the presence of anti-SARS-CoV-2 antibodies using rapid detection tests before the first dose of CoronaVac, and 28, 48, 78, 108, and 138 dpi. We found that all volunteers (21 subjects) showed anti-SARS-CoV-2 IgG at 28 dpi, and one (5%) showed specific IgM too. After 108 dpi, one volunteer showed no IgG, and 138 dpi, we found 3 volunteers present no IgG.

**Table 1:** Demographic and baseline characteristics of enrolled volunteers.

Characteristics	
Male, *n* (%)	11 (52.3)
Age, mean (range)	36 (27–61)
**Comorbidities or conditions**	
Obesity (BMI >30), *n* (%)	2 (9.5)
Metabolic conditions♠, *n* (%)	1 (4.8)
Hyperlipidemia, *n* (%)	1 (4.8)
Hypertension, *n* (%)	1 (4.8)
Cardiovascular disease, *n* (%)	0
Chronic pulmonary disease, *n* (%)	1 (4.8)
Rheumatologic disease, *n* (%)	1 (4.8)
Immunocompromised, *n* (%)	0
Allergy♣, *n* (%)	6 (28.6)
Asthma, *n* (%)	2 (9.5)
Smoker, *n* (%)	3 (14.3)

BMI, Body mass index. ♠Metabolic conditions include insulin resistance and prediabetes. ♣ Allergy considered allergic rhinitis, food allergy, and drug allergy.

### The Vaccine Triggers the Secretion of Interleukin 8

**Figure 1:**
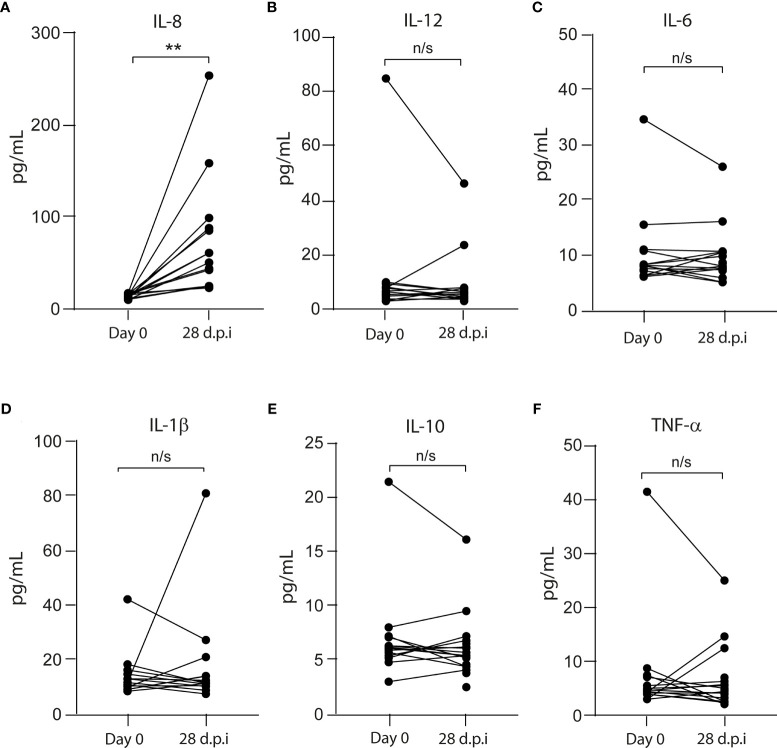
Pro- and anti-inflammatory cytokines in the plasma from CoronaVac-vaccinated volunteers. The levels of **(A)** IL-8, **(B)** IL-12, **(C)** IL-6, **(D)** IL-1β, **(E)** IL-10, and **(F)** TNF-α were measured by flow cytometry as described in *Materials and Methods*. Data represented the cytokine level on each volunteer before (Day 0) and 28 days after the second immunization (dpi). The data show individual paired values. Asterisk (**) represents significant difference (*p* < 0.01). n/s indicates non-significant differences.

The innate immune response is critical for vaccination as it activates the myeloid immune cells to release inflammatory mediators and initiate adaptive immunity the primary and post-boost immunization (9). To assess the pro- and anti-inflammatory plasma cytokine profile, we performed flow cytometry and found that, 28 dpi, only IL-8 increased its level from 14.7 ± 2.6 (on day 0) to 55.9 ± 63.8 pg/ml (on 28 dpi) (**Figure 1**). No significant changes were observed between 0 and 28 dpi in the levels of tumor necrosis factor (TNF)-α (4.7 ± 9.5 to 4.7 ± 5.1 pg/ml), interleukin (IL)-12 (6.4 ± 20.9 to 4.7 ± 9.8 pg/ml), IL-6 (8.2 ± 7.4 to 8 ± 8.6 pg/ml), IL-10 (6.2 ± 4.3 to 5.4 ± 2.7 pg/ml), and IL-1β (11.7 ± 8.5 to 11.7 ± 18.8 pg/ml) (**Figure 1**).

### CoronaVac Induces Total and Neutralizing Anti-Spike Antibodies in All Vaccinated Volunteers

**Figure 2:**
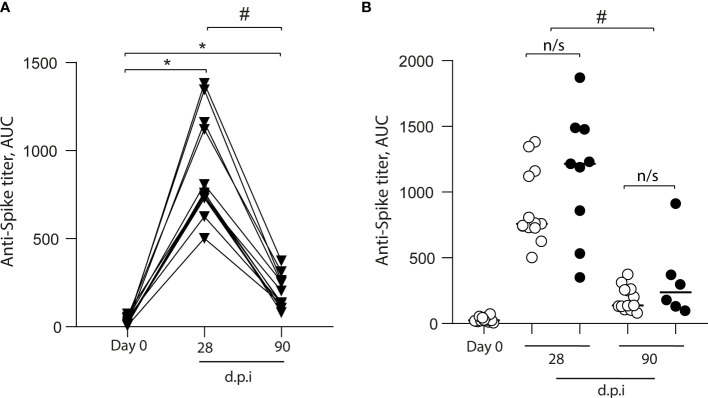
Total anti-S antibodies in the plasma of CoronaVac-vaccinated volunteers. Anti-S antibodies were quantified in plasma obtained prior to immunization (Day 0), and 28 and 90 days after the second immunization (dpi). **(A)** Antibody titers are expressed as the area under the ELISA OD curve (AUC) made from four serial dilutions (1/200, 1/400, 1/800, and 1/1600) for each volunteer (▾). The data show individual paired values. **(B)** Detail of the antibody titers for the volunteers with complete follow-up (*n* = 15) throughout the study (data from A;○), and those with no sample on some of the time points evaluated (•) were graphed and shown with median. Asterisk (*) represents statistically significant difference compared to baseline (*p* < 0.05). Hashtag (#) represents statistically significant difference between 28 dpi and 90 dpi samples. n/s, not significant (*p* < 0.05).

**Figure 3:**
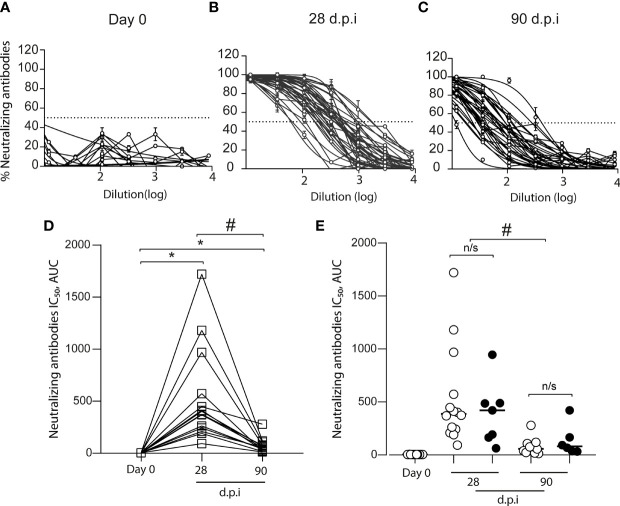
Total anti-S neutralizing antibodies in the plasma of CoronaVac-vaccinated volunteers. The neutralization curves from plasma samples of volunteers were obtained by using a pseudotyped viral particle (HIV-1–SΔ19). Samples were titrated in triplicate at serial threefold dilutions and expressed as percent of neutralization. **(A)** Total anti-S neutralizing antibodies before vaccination (Day 0). **(B)** Total anti-S neutralizing antibodies at 28 days after the second immunization (dpi). **(C)** Total anti-S neutralizing antibodies at 90 dpi **(D)** IC_50_ calculated based on inhibition rates and depicted for each sample obtained from volunteers with a complete follow-up schedule (□) (*n* = 15). The data show individual paired values. **(E)** IC_50_ calculated for the total data (*n* = 21 volunteers, including those with no complete follow-up schedule) were graphed and shown with median. Open circles (○): data from **(D)** Black circles (•): volunteers with no sample on some of the time-points evaluated. Asterisk (*) represents statistically significant difference obtained from the comparison against day 0 (*p* < 0.05). Hashtag (#) represents statistically significant difference between 28 dpi and 90 dpi samples. n/s, not significant (*p* < 0.05).

We measured the level of the anti-SARS-CoV-2 spike antibodies in 15 volunteers before and after vaccination (**Figure 2A**). We found an increase in the anti-S antibodies in all volunteers after 28 dpi (0 dpi = 24 ± 19, 28 dpi = 759 ± 278); however, after 90 dpi, the anti-S antibody levels declined (90 dpi = 137 ± 92.3), although they were higher than pre-immunization levels (**Figure 2A**). When we analyzed data from volunteers for whom samples were not always collected, the increase in anti-S antibody levels showed the same kinetics (**Figure 2B**). Additionally, we measured SARS-CoV-2 neutralizing antibodies (NAbs) using the HIV-1–SΔ19 pseudotype (8). We found no volunteers with anti-spike neutralizing response before immunization (0 dpi = 4 ± 0.7, **Figure 3A**). In contrast, after 28 and 90 dpi, we found anti-S neutralizing antibodies in all volunteers in different levels (28 dpi = 388.1 ± 454.5, 90 dpi = 57.1 ± 67.6, **Figures 3B, C**). All 15 volunteers (complete follow-up) show an increase in the IC_50_ after 28 dpi (**Figure 3D**). Furthermore, after 90 dpi, the neutralizing antibody levels declined; however, some volunteers still showed this type of antibodies. Importantly, these results are consistent with those observed for the volunteers with a not complete follow-up (**Figure 3E**).

### CoronaVac Induces Immune Cellular Response

**Figure 4:**
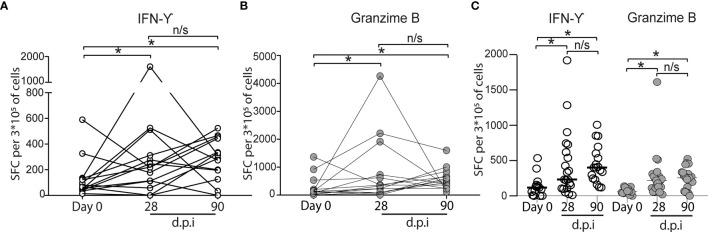
Cytokine secreting cells of vaccinated volunteers *in vitro* induced by SARS-CoV-2 S protein. **(A)** IFN-γ (○). **(B)** Granzyme (•). The number of ELISPOT-forming cells (SFC) per 3 × 10^5^ was quantitated in PBMC, obtained before vaccination (indicated as "Day 0"). Twenty-eight and 90 days after the second immunization (dpi) (*n* = 15 volunteers), the data show individual paired values. **(C)** Total data from 21 volunteers (same volunteers from **(A, B)** and others with no sample on some of the time points evaluated) were graphed and shown with median for IFN-γ (○) and Granzyme (•). Asterisk (*) represents statistically significant difference (*p* < 0.05) obtained from the comparison against Day 0. n/s indicates non-significant differences.

To explore the cellular immune responses to SARS-CoV-2 after CoronaVac vaccination, we isolated PBMCs from the 15 volunteers with complete follow-up, and the PBMCs obtained were stimulated *in vitro* with the SARS-CoV-2 spike (S) protein. We observed a significant increase in the number of IFN-γ spot-forming cells (SFCs) that specifically responded to the recombinant S protein after 28 dpi (0 dpi = 129 ± 402, 28 dpi = 343.5 ± 1,199) (**Figure 4A**). In most of the cases, IFN-γ SFCs continue to increase at day 90 dpi (90 dpi = 490 ± 384.2). Similarly, a significant increase in the number of granzyme SFCs was also observed in all, but one sample, after 28 dpi (0 dpi = 129 ± 402, 28 dpi = 256 ± 152.4) (**Figure 4B**), and most of them increased further at day 90 dpi. Altogether, these results suggest the activation and presence of CD4+ Th and cytotoxic CD8+ T memory cells, all required for an effective viral clearance (10).

## Discussion

In this study, we analyzed the immune response before immunization, 28 and 90 days after the second immunization (dpi) with CoronaVac during the current COVID-19 mass vaccination schedule applied in Chile. Notably, the results indicate that CoronaVac induces a robust humoral immune response and cellular immune memory of at least 90 dpi.

CoronaVac against SARS-CoV-2 triggers the secretion of IL-8 in all volunteers tested. In PBMC and THP-1 cells, the S protein from SARS-CoV also increased IL-8 levels (11). Similarly, SARS-CoV-2 Nsp14 protein, which contributes to the activation of NF-κB signaling, also induces the increase in IL-8 and IL-6 (12). This suggests that at least SARS-CoV-2 Spike and Nsp14 proteins can be responsible to produce IL-8 in the vaccinated volunteers. The impact of sustained higher levels of IL-8 after vaccination protocol is unclear; however, in ICU patients, IL-8 levels are predictive of survival in primo-infected patients (13).

Most of the vaccine’s protection is generated by the production of specific antibodies; however, the levels, duration, and protection of circulating antibodies vary depending on the type of pathogen and its immunogenic characteristics (14). Our data show that 100% of the volunteers participating in this study present seroconversion and neutralizing antibodies production after 28 and 90 dpi. The induction of neutralizing antibodies after CoronaVac administration in a 28-day interval between the first and second dose is consistent with the data reported in the phase 3 trial of CoronaVac conducted in Chile, which showed over 90% seroconversion for neutralizing antibodies after 42 dpi (4). Neutralizing antibodies are critical for vaccine efficacy in preventing infection since robust neutralizing-antibody responses appear to be more protective than those with weaker response (15). Although in our current study the neutralizing antibodies wane after 3 months of the second dose of CoronaVac, further studies and characterization of memory B and plasma cells are needed to associate this condition with a potential lack of protection against COVID-19.

A robust antibody response is insufficient to avoid severe disease (16) and the cellular immune response is also required for an effective viral clearance (10). Here, results show that CoronaVac induced a cellular response against the S protein of SARS-CoV-2. The increase in IFN-γ and Granzyme B-producing cells after antigen stimulation suggests the activation of CD4^+^ Th cells and cytotoxic CD8^+^ T memory cells. The presence of T cell-mediated response after CoronaVac vaccination concurs with BNT162b1 vaccine, which elicited robust CD4^+^ and CD8^+^ T-cell responses until 43 dpi (17). The ability of CoronaVac to activate a cellular immunity is relevant since CD4^+^ and CD8^+^ T-cell-mediated response was significantly lower in severe/critical patients in comparison to those with mild and mid-grade groups, data that strongly support the participation of T cells in the immune protection against COVID-19 (18).

In summary, our analysis showed that two dose immunizations with CoronaVac vaccine generates neutralizing antibodies, IFN-γ-producing cells, and Granzyme B 28-days after the second immunization. Moreover, the increase in Granzyme B was sustained after 90 days of the second immunization. It is of great importance for public health to determine over time the levels of neutralizing antibodies, cellular immunity, and the dynamic of memory response to SARS-CoV-2 in the population after vaccination to determine the need for modifications in the immunization protocols, application of a third/booster dose with the same or a different vaccine, and improvement of future vaccines.

## Data Availability Statement

The raw data supporting the conclusions of this article will be made available by the authors, without undue reservation.

## Ethics Statement

The studies involving human participants were reviewed and approved by Ethics Committee, Universidad de Santiago de Chile (172/2021). The patients/participants provided their written informed consent to participate in this study.

## Author Contributions

ELISPOT and cell isolation: AE, HP, JG, and CB-A. Antibody neutralization assays: MA, LA-P, FV-E, and RS-R. Total antibody and cytokine analysis: MB, LV, and VS. S protein production: CV, JH, and LC. Study coordination: DV. MNS: EN-L. MNS and study design. AE, FER-L, AMS, MI, and CA-C. All authors contributed to the article and approved the submitted version.

## Funding

The authors thank the support of ANID-COVID1005 (EN-L), ANID-COVID1038 (AMS, FER-L, MI, CA-C), FONDECYT Postdoctoral fellowship N° SECTEI/138/2019 (LA-P), FONDECYT grants 1211547 (FV-E), 1211841 (FER-L), 1201240 (CV, JH, and LC), 1201664 (MI, AMS, and FER-L), 1181814 (EL-S), 1190156 (RS-R), and 1180666 (AE); DICYT 021943AC (CA-C), SOFOFA SiEMPRE 18 CTBT-102728 (CV, JH, and LC). The funding bodies were not involved in the study design, collection, analysis and interpretation of data, the writing of this article, or the decision to submit results for publication.

## Conflict of Interest

The authors declare that the research was conducted in the absence of any commercial or financial relationships that could be construed as a potential conflict of interest.

## Publisher’s Note

All claims expressed in this article are solely those of the authors and do not necessarily represent those of their affiliated organizations, or those of the publisher, the editors and the reviewers. Any product that may be evaluated in this article, or claim that may be made by its manufacturer, is not guaranteed or endorsed by the publisher.
